# A Review of the Utility and Limitations of Artificial Intelligence in Retinal Disorders and Pediatric Ophthalmology

**DOI:** 10.7759/cureus.71063

**Published:** 2024-10-08

**Authors:** Kristie M Labib, Haider Ghumman, Samyak Jain, John S Jarstad

**Affiliations:** 1 Department of Ophthalmology, University of South Florida Health Morsani College of Medicine, Tampa, USA

**Keywords:** artificial intelligence, machine learning, ophthalmology, pediatric ophthalmology, retina

## Abstract

Artificial intelligence (AI) is reshaping ophthalmology by enhancing diagnostic precision and treatment strategies, particularly in retinal disorders and pediatric ophthalmology. This review examines AI's efficacy in diagnosing conditions such as diabetic retinopathy (DR) and age-related macular degeneration (AMD) using imaging techniques, such as optical coherence tomography (OCT) and fundus photography. AI also shows promise in pediatric care, aiding in the screening of retinopathy of prematurity (ROP) and the management of conditions, including pediatric cataracts and strabismus. However, the integration of AI in ophthalmology presents challenges, including ethical concerns regarding algorithm biases, privacy issues, and limitations in data set quality. Addressing these challenges is crucial to ensure AI's responsible and effective deployment in clinical settings. This review synthesizes current research, underscoring AI's transformative potential in ophthalmology while highlighting critical considerations for its ethical use and technological advancement.

## Introduction and background

The field of artificial intelligence (AI) can be traced back to the year 1955 by John McCarthy and his colleagues who formally established the field. Their objective was to create an interdisciplinary domain that explored the science and mathematics of making intelligent machines [1]. In the human brain, information is continually processed and organized through the growth of neural networks as it continues to learn new information. AI is founded on this principle, where created neural networks may not only store information but also enhance their ability to organize and generate concepts through the training of AI models [1].

AI has a growing influence in the field of medicine, as with many recent virtual AI models transforming the healthcare landscape. Virtual AI systems are computational networks designed to identify, quantify, or organize medical information when provided with patient information. AI systems vary in their training methods and type of output data. Many AI systems are trained by machine learning or deep learning (DL), which are methods that incorporate artificial neural networks (ANNs) or convoluted neural networks to generate outputs. The idea is that these systems can replicate the functioning of the human brain and "learn" with exposure to data, therefore improving the accuracy of outputs. The learning process of AI systems includes unsupervised learning, supervised learning, or reinforcement learning. Unsupervised learning is learning by recognizing patterns when exposed to data. Supervised learning is exposing networks to pre-identified characteristics that the system learns to identify. Reinforcement learning involves learning through reinforcements or punishments. Ultimately, these trained AI models have demonstrated their effectiveness in improving ophthalmological care.

There is a notable rise in AI integration across various medical specialties, but ophthalmology stands out as particularly well-suited for AI adoption. This is primarily due to its heavy reliance on imaging and data for tasks such as the screening, diagnosis, prognosis, and treatment of eye conditions. The myriad of imaging modalities utilized in ophthalmology offers plenty of opportunities for the development of AI programs using imaging data. AI's involvement in ophthalmology is groundbreaking, with the possibility of enhancing diagnostic precision, enabling early disease direction, and augmenting the overall quality of care. Numerous recently developed AI models have demonstrated their efficacy in ophthalmological care, further substantiating the transformative impact of AI in ophthalmology. AI will likely play an increasingly pivotal role in ophthalmology, given its recent advancements in ophthalmological care. AI models are anticipated to be the future of ophthalmology by revolutionizing the management of eye conditions. The purpose of this study is to comprehensively review the current literature regarding the utility of AI within the ophthalmology specialties of the retina and pediatrics. Furthermore, we hope to elucidate current limitations surrounding the use of AI and potential solutions to these concerns.

## Review

Artificial intelligence and the retina

AI and Retinal Disease Diagnosis

Artificial intelligence has made remarkable strides in the detection, prognosis, and treatment management of retinal diseases, which are among the leading causes of vision loss and blindness worldwide [2]. Many AI systems have similar functionality to ophthalmologists, which is demonstrated by their reliance on high-quality images to accurately identify retinal pathology. Like human ophthalmologists, AI requires clear images to properly examine all features of the retina. Otherwise, small pathological characteristics may go unnoticed [3].

Similar to human ophthalmologists, retinal pathology detection is stronger when presented with complete images of the retina compared to their performance with an analysis of partial retinal images. Further, the limitations of AI system proficiency are also compared to those of trained retina specialists. This is demonstrated by the finding that AI generally detects more overt pathologies with higher accuracy, such as moderate-sized hemorrhages, compared to more subtle pathological findings, such as microhemorrhages [4].

One common application of AI in retinal disease detection is the analysis of images from color fundus photography (CFP). AI algorithms are developed to automatically detect abnormal pathological biomarkers associated with retinal diseases [5]. This is achieved by training the AI system to recognize the locations and characteristics of anatomical landmarks in healthy retinas. Therefore, when the system is presented with images containing abnormal retinal anatomy, then it may automatically identify them.

AI systems designed for disease detection function examine the retinas in a similar manner to trained retina specialists. Initially, they identify key landmarks, such as the optic nerve head, fovea, and macula. The localization of the main retina landmarks then assists with identifying any abnormal retinal characteristics [6]. This examination method is beneficial so that AI systems may identify subtle abnormalities such as scant microaneurysms that may be easily missed in an otherwise healthy retina [7].

AI and Age-Related Macular Degeneration (AMD)

Age-related macular degeneration (AMD) is one of the leading causes of vision loss in adults worldwide, and AI has enhanced its diagnosis. AI programs have been created utilizing images obtained through optical coherence tomography (OCT), a cutting-edge imaging modality widely used to capture detailed images of the retina. OCT is a gold standard noninvasive imaging tool used for the screening and diagnosis of prevalent retinal biomarkers of disease such as choroidal neovascularization or choroidal edema [5,8]. OCT is unique in that it identifies subtle retinal abnormalities that cannot be identified by slit lamp or fundoscopic examination. For macular degeneration, OCT can pick up hyperreflective foci and can capture outer retinal thinning, which are indicators of early age-related macular degeneration detection on imaging that may be unnoticed on fundoscopic examination. Furthermore, OCT detecting macular degeneration prior to symptom onset/progression allows it to be a valued screening tool.

An additional biomarker for AMD that OCT detects is drusen, which is defined as yellow deposits composed of cellular debris, protein, and lipids. AI models can determine the dimensions and volume of drusen deposits using OCT images, which cannot be effectively measured through fundoscopic examination [5]. These measurements are beneficial as they may demonstrate the extent of progression of AMD. For instance, it has been found that a rapid reduction of drusen volume is strongly correlated with a late-onset AMD [9]. Therefore, the combination of AI programs and state-of-the-art abilities has immense potential in revolutionizing the management of retinal diseases.

AI and Diabetic Retinopathy (DR)

With the considerable prevalence of diabetic retinopathy, AI stands to play a key role in its diagnosis and management. In 2021, 9.6 million people had diabetic retinopathy, which comprises 26% of people with diabetes; 1.84 million people had vision-threatening diabetic retinopathy, which comprises 5.06% of people with diabetes [10]. Currently, there are devices in development that may screen for diabetic retinopathy and classify disease based on severity. The IDx-DR device was recognized as the first device to be US Food and Drug Administration (FDA)-approved to screen for diabetic retinopathy [11]. The device first evaluates the quality of fundoscopic images to ensure all areas are clear and sharp. Its algorithm then scans images for pathological characteristics indicative of diabetic retinopathy, such as microaneurysms and exudates. Finally, it classifies disease into categories, ranging from no DR to moderate or severe vision-threatening DR. These devices are used in the office, but the development of smartphone devices to aid in early DR detection is ongoing.

AI in pediatric ophthalmology

Barriers to Optimal Care in Pediatric Ophthalmology

The diagnosis, presentation, and treatment of ophthalmological disease differ substantially in children compared to adults [12]. As there is currently a shortage of pediatric ophthalmologists, AI may help optimize patient care enhancing the efficiency of pediatric eye disease management [13].

Notable strides have been made in the AI implementation of adult eye disease management, but there is much less progress in AI in the realm of pediatric ophthalmology despite the need [12]. The combination of AI and care delivered by pediatric ophthalmologists may improve efficiency in healthcare delivery. The practice of pediatric ophthalmology may be at times more challenging compared to adult ophthalmology. Challenging factors include physical examination difficulties with young children exhibiting uncooperative behavior and short attention spans, having poorly dilating pupils, or demonstrating noncompliance with placing eye drops [14,15].

The management of visual development and eye growth is essential during a child's early years to prevent permanent visual deficits. With many barriers impacting the efficient and accurate diagnosis of eye conditions in pediatric ophthalmology, AI tools may allow for standardized and objective methods of eye disease management.

AI in Retinopathy of Prematurity (ROP)

A major milestone in the integration of AI in pediatric ophthalmology is the ability to screen for retinopathy of prematurity, a leading cause of vision loss in neonates [12]. Automated methods of diagnosis not only provide an objective measure of diagnosis but also offer a less challenging alternative for retina examination in neonates. More traditional methods include the use of indirect ophthalmoscopy and neonatal screening programs.

Recent approaches to AI in ROP diagnosis include using convolutional neural networks (CNNs) to identify dilated and tortuous vasculature on color fundus photographic images, which is a characteristic sign of ROP [16]. AI has demonstrated the ability to detect disease with accuracy levels similar to ROP experts. An example of such a program is the i-ROP system, which has been found to identify ROP versus no ROP in retinal images with 95% accuracy, with ROP experts obtaining accuracies ranging from 92% to 96% and nonexperts with an average accuracy of 81% [17]. i-ROP was found to effectively stage ROP by identifying images with vascular severity scores from a 1-9 scale, which correlated with the clinical assessment of disease progression, which was determined by the zone, stage, and extent of ROP disease (p<0.001) [18]. Therefore, current AI tools may enhance the diagnosis of ROP.

AI in Pediatric Cataracts

The diagnosis and treatment of cataracts in pediatrics present unique challenges compared to adults. The diagnosis requires a thorough slit lamp examination, which may be difficult due to some children being unable to remain still during the examination. Additionally, some children with intraocular cataracts may also have other anatomical abnormalities that may impede the quality of the examination [19]. To address these challenges, the CC-Cruiser is a program that aids in the diagnosis and treatment of cataracts. Fundoscopic images are first uploaded to the CC-Cruiser platform. The software then identifies the lens and determines whether a cataract is present [19,20]. It also identifies the characteristics of the cataract, such as location, size, and density, and then offers treatment recommendations for the cataract [19,20].

In a multicenter-recognized control trial, the platform was found to be less accurate with cataract diagnosis and treatment recommendation compared to cataract experts. However, it did receive a high level of patient satisfaction due to the efficiency of the tool and less time consumption during diagnosis [20]. It was considered that the lower accuracy of the tool may be due to poor-quality images affecting analysis. The quality of images may have been affected by moving patients, obscuring eyelashes or eyelids, or image-obscuring reflections [20]. Therefore, while AI tools such as the CC-Cruiser show promising results in pediatric cataract management, further research on the tool is required.

AI in Strabismus

AI exhibits the potential to assist with strabismus diagnosis and treatment planning. As of 2020, the global prevalence of strabismus ranged from 0.14% to 5.65%. Without proper intervention, strabismus may cause amblyopia [21], a lack of stereopsis, reduced academic performance [22], and delayed developmental milestones [23].

One avenue of AI application in the diagnosis of strabismus is through the development of mobile applications. An example of such is an artificial intelligence application developed utilizing a CNN with efforts to diagnose strabismus less subjectively and more efficiently than standard methods. The results demonstrate the potential for the application to supplement standard clinical methods, but further research is required [24].

Further, an AI platform was developed with three DL systems for strabismus diagnosis, angle evaluation, and operative planning based on corneal light reflection photos. The AI platform achieved high accuracy, which may serve as a helpful tool for strabismus management [25]. Although AI directs strabismus management in the right direction, further research is required.

AI limitations

Ethical Dilemma

AI has been demonstrating increasing accuracy with its abilities in ophthalmology, ranging from improved diagnostic measures to creating personalized therapies for patients. However, significant questions still arise as to the potential ethical implication increased AI development could hold [26]. The continual development of AI algorithms may carry the potential for error and inherent bias, issues that may hold a large degree of influence due to the sensitive nature of AI's responsibilities. These errors could also occur across racial and economic lines, leading to a loss in equity and fairness [27]. Significant problems in the creation of the algorithm can lead to overfitting, a problem in which there is a projected accuracy higher than that seen in reality. The use of smaller data sets in training can have major ethical impacts on the lives of patients [26]. The lack of human connection in AI usage has also been the cause of ethical concern. Ophthalmology-based practices rely heavily on the doctor-patient relationship, with the use of AI leading to a more robotic relationship without the involvement of necessary human connection in patient care. Both physicians and patients have stressed potential concerns about the way AI could change the doctor-patient relationship and remove the ability to provide informed advice and sound decision-making [28].

Solutions to the ethical dilemmas presented by AI revolve around the algorithm and better framework around AI usage. The use of data sets is key to the accuracy of AI, but ensuring the best data sets possible can lead to the most accurate results and the reduction of ethical problems. Involving large, targeted data sets can ensure that the AI is well-trained for the specific ophthalmology-based purpose [26]. The overall legislation and oversight of these algorithms can also be beneficial in increasing ethical standing. The FDA approval and monitoring of AI algorithms have been largely beneficial in ensuring a standard is maintained for AI alongside boosting confidence in the use of AI. Continual oversight can ensure that AI is developed according to international and national standards [26,29]. Further success can be achieved by frameworks of an interdisciplinary nature on the usage of AI in healthcare, specifically ophthalmology. Alongside the input of physicians, the involvement of philosophers and ethicists can ensure positive, detailed frameworks that allow AI to be used effectively and ethically [27]. As AI continues to progress, questions will continue to arise as to the fair and just nature of AI, with the answers lying in continual improvement and change [28,30].

Loss of Privacy

The careful balance between privacy and effective AI usage is necessary to understand the potential AI holds. Questions regarding what is classified as patient privacy versus information necessary for patient health need to be answered prior to the further development of AI in ophthalmology [31]. Privacy in AI largely revolves around preventative measures against unwanted intrusions, helping to guard patient information against hacks and leaks [32]. The rampant use of malware for the purposes of securing personal information has decreased trust in AI algorithms, with patients wary about the potential belief in AI and the level to which their information can be guarded. This risk has steadily increased over the years, with more information including facial identity now being taken in the process of patient care in healthcare and ophthalmology [33]. Current protections for privacy largely revolve around important legislation to protect patients. The Health Insurance Portability and Accountability Act (HIPAA) in the United States provides a great deal of protection for the way patient information can be used, allowing for confidence in the medical system. The legislation classifies certain patient information as protected from unjust use, with only certain exceptions for the way it can be used for the purposes of ensuring patient health. State protections also increase privacy for patients, helping to monitor on a state-by-state basis [31,34].

The recent privatization of AI in healthcare has also been implicated as a cause of decreased privacy. This loss of central oversight has led to decreased algorithm quality, which is harmful to patient protection [35]. Focusing on patient rights and how they can be continually protected through the increased presence of artificial intelligence is necessary in ophthalmology and in overall healthcare systems [32]. The use of increased data protections, strict oversight on potential AI uses, and reformed legislation can potentially boost patient privacy. Analysis has revealed that current protection standards do not account for the changes seen by AI involvement, meaning change is needed for protection to be reimplemented [33]. Privatization in AI is important for further progress, but protections are stifled in the name of advancement and profit. Ensuring that strict measures are placed and enforced on the way corporations can build and utilize patient data for AI purposes can help mitigate the problems caused by the lack of government control [35].

Data Set Development

The quality of AI algorithms is largely tied to the specific level of training through which the algorithm is exposed. The quality and quantity of the data sets used correlate to the accuracy of the AI and the capability of the AI to mark significant factors in the patient care pathway [36]. Current problems with effective data set development have led to a domino effect, impacting the quality of AI for the purposes of diagnosis and treatment. Missing and unspecified data has produced small and ineffective sets of data in comparison to the level of data sets needed to produce patient and physician confidence [37]. Alongside the lack of data, the cost of data production has led to problems with data set creation. The cost associated with image production has limited the availability of images and the locations with imaging capabilities. Even as these images are taken, the process through which they are inputted into neural networks can lead to significant problems [38]. The image size itself severely inhibits images from being used for the purpose of training neural networks, with the perfect combination of image size and neural network needed for effective information transfer. The lack of labels further hampers development, ensuring that the images do not have the proper classifications needed to create a well-developed set of data [38].

The development of data sets requires the use of additional solutions rather than just the creation of original data points. The development of synthetic, or artificial, data points could help to increase data set progression as continual advancements for the collection of original data are being made [36]. Processes including data augmentation can allow for the development of new points from the existence of smaller data sets, helping to expand available training data [36]. Biased data sets also hold a great deal of value, holding potential data points from which more functional, non-biased data sets can be built. To help repurpose these data points, processes including downsampling can be used to help salvage and rebuild data sets without the influence of biased or skewed data [37]. The research side of ophthalmology can also largely benefit from the development of well-organized data sets. AI holds the potential to unlock major advances in research, leading to a major development in medical and ophthalmology-based understanding. The use of large-scale computing can help to organize data rather than have the potential for human error, with the development of developed data sets through which AI can derive clear and accurate results [36].

Hidden and Unclear Algorithm

Although knowledge regarding the potential uses and applications of AI has continued to rise, the way AI operates and the various ways it proceeds to the resulting outcome have resulted in the use of "black box" terminology [39]. The manner in which the training occurs and the specific data sets used remain hidden from those who utilize the AI, decreasing understanding and confidence in the AI's abilities. In healthcare itself, physicians have voiced doubts as to the lack of understanding regarding the system, as well as the decreased reliability due to its hidden nature [40]. The other side of this argument revolves around the intricacies through which the AI is developed and trained, being too complicated for general understanding and developing the notion of a "black box" [39]. The specific nature through which training occurs could help to largely boost confidence and open the concept of AI into ophthalmology. Artificial neural networks (ANNs) contain self-learning features central to development, with an explanation of how this occurs lending a great deal of confidence to the physicians who use these systems in the clinic. The development and installation of other software can also help to simplify this process, creating the necessary transparency [41]. Current studies have shown the benefits of the use of an explained versus an unexplained AI system. Through better understanding, ophthalmologists are able to better utilize the technology to maximize efficacy and better understand in which situations the AI system can provide the best results [40].

## Conclusions

Artificial intelligence has demonstrated significant potential in enhancing the diagnosis and management of retinal disorders. By leveraging advanced imaging techniques, such as optical coherence tomography (OCT) and color fundus photography, AI algorithms can detect pathologies such as diabetic retinopathy and age-related macular degeneration with high precision. These systems replicate the diagnostic processes of ophthalmologists, identifying critical anatomical landmarks and abnormalities with remarkable accuracy. The integration of AI in retinal care is a promising advancement, offering the potential for more accurate, efficient, and standardized diagnostic processes. In pediatric ophthalmology, AI's role is equally transformative, particularly in addressing the unique challenges of diagnosing and managing eye conditions in children. The shortage of pediatric ophthalmologists and the difficulties associated with examining young patients underscore the need for AI-driven solutions. AI systems can assist in screening for conditions, such as retinopathy of prematurity (ROP) and pediatric cataracts, providing objective and efficient diagnostic support.

Despite the promising applications of AI in ophthalmology, several limitations and ethical concerns need to be addressed. The accuracy of AI algorithms depends heavily on the quality and diversity of the training data sets, which, if biased or insufficient, can lead to erroneous diagnoses and exacerbate health disparities. Additionally, the "black box" nature of AI systems, where the decision-making process is not transparent, raises concerns about trust and reliability among healthcare providers and patients. Privacy issues also emerge, as the use of patient data for training AI models must comply with stringent regulations to protect patient confidentiality. Ethical dilemmas regarding the potential loss of human connection in patient care and the need for continual oversight and legislation to ensure responsible AI deployment further complicate its integration into clinical practice. Addressing these challenges through improved data quality, transparency, and ethical frameworks is crucial for the sustainable and equitable adoption of AI in ophthalmology.
